# The role of transposable element clusters in genome evolution and loss of synteny in the rice blast fungus *Magnaporthe oryzae*

**DOI:** 10.1186/gb-2006-7-2-r16

**Published:** 2006-02-28

**Authors:** Michael R Thon, Huaqin Pan, Stephen Diener, John Papalas, Audrey Taro, Thomas K Mitchell, Ralph A Dean

**Affiliations:** 1 Department of Plant Pathology and Microbiology, Texas A&M University, College Station, TX 77843, USA; 2 Center for Integrated Fungal Research, North Carolina State University, Raleigh, NC 27695, USA

## Abstract

**Background:**

Transposable elements are abundant in the genomes of many filamentous fungi, and have been implicated as major contributors to genome rearrangements and as sources of genetic variation. Analyses of fungal genomes have also revealed that transposable elements are largely confined to distinct clusters within the genome. Their impact on fungal genome evolution is not well understood. Using the recently available genome sequence of the plant pathogenic fungus *Magnaporthe oryzae*, combined with additional bacterial artificial chromosome clone sequences, we performed a detailed analysis of the distribution of transposable elements, syntenic blocks, and other features of chromosome 7.

**Results:**

We found significant levels of conserved synteny between chromosome 7 and the genomes of other filamentous fungi, despite more than 200 million years of divergent evolution. Transposable elements are largely restricted to three clusters located in chromosomal segments that lack conserved synteny. In contradiction to popular evolutionary models and observations from other model organism genomes, we found a positive correlation between recombination rate and the distribution of transposable element clusters on chromosome 7. In addition, the transposable element clusters are marked by more frequent gene duplications, and genes within the clusters have greater sequence diversity to orthologous genes from other fungi.

**Conclusion:**

Together, these data suggest that transposable elements have a profound impact on the *M. oryzae *genome by creating localized segments with increased rates of chromosomal rearrangements, gene duplications and gene evolution.

## Abstract 2

Analysis of the *Magnaporthe oryzae *chromosome 7 and comparison with syntenic regions in other fungal genomes suggests that transposable elements create localized segments with increased rates of chromosomal rearrangements, gene duplications and gene evolution.

## Background

*Magnaporthe oryzae*, a member of the *M. grisea *species complex [1], causes blast disease of rice and is one of the most destructive pathogens of this important food crop [2]. Its recently published genome sequence [3] is the first for a plant pathogenic filamentous fungus and is providing new insight into the molecular and genetic basis for pathogenesis. *M. oryzae *shares a number of traits with other plant pathogenic fungi, such as the formation of specialized infection structures called appressoria that are important in penetrating the plant epidermis. *M. oryzae*, along with many other plant pathogens, also exhibits a genetically controlled pattern of host recognition, called the gene-for-gene interaction, that mediates host range [4]. Because of the commonality of these features among plant pathogenic fungi, its genetic tractability, and its economic importance, *M. oryzae *has been developed as a model for studying infection related morphogenesis and fungal-plant interactions [5].

The genome of *M. oryzae*, like that of many living organisms, is rich in repetitive DNA. Analysis of the whole genome shotgun sequence (WGS) suggests that more than 9.7% of the genome is made up of repetitive DNA, a significant portion of which is derived from transposable elements (TEs) [3]. TEs have had an important impact on the genome and TE insertions are known to cause mutations in genes that mediate host range [6-8]. Previous studies have shown that TEs are arranged in distinct clusters in the genome [9-14]. Clustered TE distribution has been reported in many organisms, though in most cases the actual mechanisms leading to this distribution are unknown. In many species, including *Drosophila melanogaster *[15], *Arabidopsis thaliana *[16], and *Tetraodon nigroviridis *[17], TEs tend to accumulate in heterochromatic regions of the chromosomes, leading to a negative correlation between genetic recombination rate and TE density. The most common explanation for this is that selection against the deleterious effects of TEs is weaker in chromosomal regions with lower recombination rate, although exceptions to this model are also known. In *D. melanogaster*, TEs are preferentially clustered in regions with low recombination rate [18], while in *C. elegans*, a positive correlation between recombination rate and DNA transposons has been observed [19]. Clearly, the selection model is insufficient to explain the distribution of TEs in all cases.

A prior study of one of the first full length sequences of a bacterial artificial chromosome (BAC) clone from the *M. oryzae *genome showed that this approximately 100 kb segment shared a considerable amount of synteny with *Neurospora crassa *and opens the possibility that more extensive conservation of synteny may exist between these species [20]. This conservation of synteny is unexpected, since it is well known that *M. oryzae *isolates have extremely variable karyotypes due, at least in part, to the presence of transposable elements [11,21]. Estimates of divergence dates within the ascomycetous fungi suggest that *M. oryzae *and *N. crassa *may be separated by more than 200 million years of divergent evolution [22]. The ancient radiation of these fungi and the karyotype variability do not fit the expectation that large segments of conserved synteny should exist between *M. oryzae *and other species.

The availability of whole genome sequences for *M. oryzae *[3], *N. crassa *[23] and several other filamentous fungi are now allowing for more in-depth analyses of the organization of repetitive elements and their role in the evolution of fungal genomes. Recently, we completed a draft sequence of 38 BAC clones spanning chromosome 7 from *M. oryzae*, which we combined with selected contigs from the *M. oryzae *WGS, to yield a new chromosome 7 sequence assembly that was 4 Mb in length and contained 50 gaps. We used this sequence to measure the extent of conserved synteny between *M. oryzae *and the genome sequences of *N. crassa*, *Fusarium graminearum *and *Aspergillus nidulans*. We found that large segments of conserved synteny could be identified between these fungi and that syntenic blocks were negatively correlated with clusters of TEs. In addition, we found that chromosomal regions containing TE clusters also have higher rates of gene duplications and rates of gene evolution.

## Results

### Content and distribution of repetitive DNA

**Figure 1:**
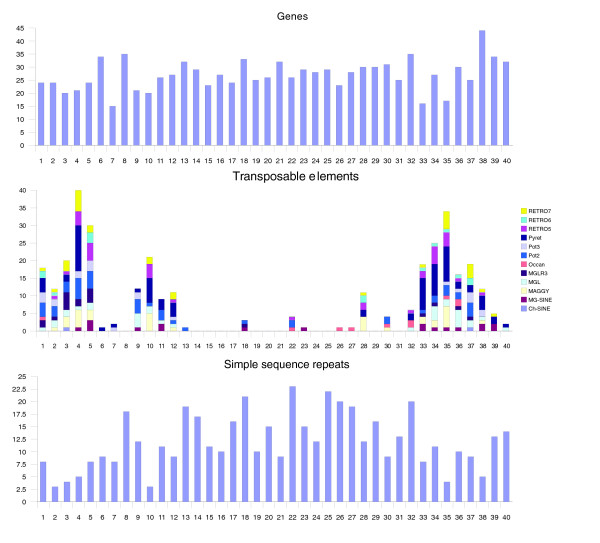
Distribution of sequence features on chromosome 7. Chromosome 7 was divided into non-overlapping 100 kb segments. The vertical axis of each chart represents the number of features per segment after correcting for gaps in the sequence. Only the three most abundant transposable elements are shown.

The combined BAC-WGS sequence of chromosome 7 is 3,997,066 base-pairs (bp) in length, including fifty 200 bp gaps. The centromere has been genetically mapped to a region between markers CH5-75H and cos156 (Figure 1; M Farman, personal communication). This region also contains a single gap, which probably represents the centromere, since centromere-like sequences were not found in the flanking BAC sequences. The sequence ends are estimated to be less than 40 kb from the telomeres, based on the presence of telomeric repeats found within the fosmid end sequences that make up the WGS (M Farman and C Rehmeyer, personal communication). Using a curated set of TE reference sequences, we scanned the chromosome 7 sequence with RepeatMasker [24]. We determined that nearly 14% of chromosome 7 is composed of repetitive DNA, considerably more than the estimate of 8.2% that we obtained from the WGS alone. This increase of nearly 6% can be attributed to the increased sequence coverage that was derived from the BAC sequences, as well as improvements to the assembly through manual editing. Virtually all of the repetitive DNA found on chromosome 7 was in the form of TEs, with simple sequence repeats and other types of repeats comprising less than 1% of the total repetitive DNA content. In terms of number of elements, diversity (number of families) and overall contribution to the sequence, long terminal repeat (LTR) retrotransposons are the most common class (Table 1). At least 7 families of LTR type retrotransposons exist in the *M. oryzae *genome, all of which are found on chromosome 7 and make up 8.8% of the sequence.

**Table 1:** Transposable element content of chromosome 7

Transposable element	Number of elements*	Total bp length (% of chromosome sequence)
Class I (retrotransposons)		
LTRs		
MAGGY	34	89,157 (2.23%)
Pyret	79	97,918 (2.45%)
RETRO5	33	58,888 (1.47%)
RETRO6	14	30,831 (0.77%)
RETRO7	30	47,867 (1.20%)
MGLR3	19	10,967 (0.27%)
LINEs		
MGL	32	90,563 (2.27%)
SINEs		
MG-SINE	18	8,200 (0.21%)
Ch-SINE	2	1,319 (0.03%)
		
Class II (DNA transposons)		
Pot2	48	71,240 (1.78%)
Pot3	21	26,761 (0.67%)
Occan	10	16,541 (0.41%)
		
Miscellaneous or unclassified elements		
5SrRNA	5	595 (0.01%)
MGSR2	8	8,315 (0.21%)

*Includes full-length elements as well as fragments. LINE, long Interspersed repeat element; SINE, short interspersed repeat element.

We analyzed the distribution of TEs in chromosome 7 by defining non-overlapping 100 kb intervals across the sequence and measuring repetitive sequence content as the number of TEs per interval, and normalizing for gaps. A histogram of these data clearly indicates the presence of three clusters on chromosome 7 (Figure 1), containing both class I (retrotransposons) and class II (DNA transposons) elements. The presence of TE clusters on chromosome 7 is consistent with our earlier report of TE clustering based on analyses of BAC end sequences and the *M. oryzae *physical map [10], in which TE clusters were identified on all seven chromosomes. TEs are frequently reported to be associated with both telomeres and centromeres, although gaps in the combined chromosome 7 sequence prevent analyses of these regions. However, the analysis presented here indicates that TEs, in addition to being found in telomeric and centromeric regions, are also found in abundance in other parts of the chromosome. No such clustering of simple sequence repeats (SSRs) was evident, although a slight depression in SSR frequency was observed within TE clusters (Figure 1). Likewise, a slight depression in gene content was evident in regions of high TE content, which may be a result of displacement of SSRs and genes by TEs.

### TE clusters are correlated with recombination rate

**Figure 2:**
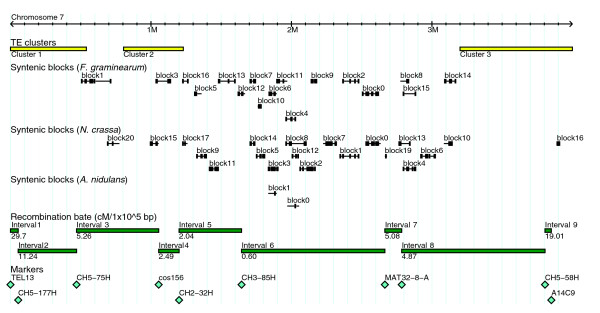
Map of chromosome 7 showing relative locations of genetic markers and other features. Recombination rate between markers is expressed as centiMorgans per 100 kb. The locations of blocks of conserved synteny, recombination rate, TE clusters, and restriction fragment length polymorphism markers are shown.

**Figure 3:**
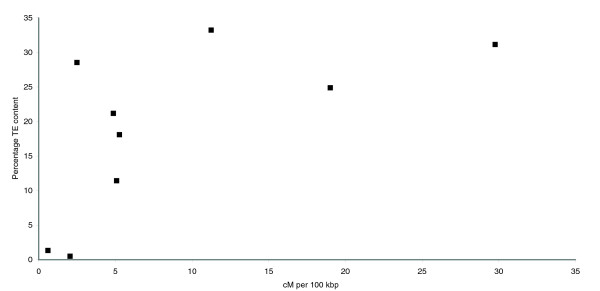
Correlation between TE content and recombination rate. Nine intervals on chromosome 7 were defined using 10 genetic markers. The Spearman rank correlation coefficient (*R*_*s *_= 0.78) was significant (*P *= 0.02) at the 95% confidence interval.

Ten markers from the anchored genetic-physical map could be unambiguously assigned positions on the BAC-WGS sequence and were used to define nine intervals spanning the chromosome (Figure 2). Recombination rate, expressed as centiMorgans per 100 kb, ranged from 0.59 to 29.7 and generally increased towards the distal ends of the chromosome arms. Centromeres are known to have greatly depressed recombination rates in many species [25,26] and we expected that the centromere would map to the segment with the lowest recombination rate. However, interval 6 has the lowest recombination rate, but the centromere is mapped to interval 3 between markers CH5-75H and cos156 (M Farman, personal communication). It is likely that recombination rate depression in the vicinity of the centromere is a highly localized effect, and not evident in the coarse scale of the genetic map. Transposable element content was expressed as the percentage of DNA derived from TEs identified with RepeatMasker. Using a Spearman rank correlation test, we determined that there is a significant (*P *= 0.02) positive correlation between chromosome recombination rate and TE content (Figure 3). To determine whether this pattern was representative of the whole genome, we repeated the test using the WGS. Of the 119 genetic markers that were anchored to the WGS, 64 were removed from the analysis because they were anchored to multiple locations in the genome or because the order of markers on the physical map was not consistent with the genetic map. The remaining markers were used to define 54 intervals on all seven chromosomes. Using this data set, we also found a significant (*P *= 0.0003) positive correlation between recombination rate and TE content, indicating that the pattern of TE distribution observed in chromosome 7 is representative of the whole genome.

### Blocks of conserved synteny

The FISH software package provides a fast algorithm to identify chromosomal segments with conserved synteny and includes a statistical evaluation of the segments [27]. Using FISH, we identified 21 syntenic blocks (*P *< 0.001) between chromosome 7 and the *N. crassa *genome that ranged in size from 5 to 16 orthologous gene pairs (Table 2). The largest block consists of 16 conserved gene pairs, and spans 86,053 bp of chromosome 7, a region that includes 30 predicted genes. The remaining 14 genes in the segment either did not have identifiable homologs in *N. crassa *or had homologs to genes that were not identified as members of the syntenic block. Interestingly, all of the blocks were found on *N. crassa *chromosome 1, though the relative order of the syntenic blocks was not retained between the two chromosomes. We found similar patterns of conserved synteny when we used the same analytical methods to compare chromosome 7 to two other filamentous fungi. Seventeen blocks were identified between chromosome 7 and the *F. graminearum *genome, 14 of which were found on chromosome 2. The remaining three were found on chromosome 4, suggesting either a translocation of a large chromosomal segment in the *F. graminearum *lineage or an error in the genetic map. Only two syntenic blocks were identified in *A. nidulans*, reflecting its greater evolutionary distance to *M. oryzae*.

**Table 2:** Syntenic blocks containing 5 or more orthologous gene pairs between chromosome 7 of *M. oryzae *and related fungi

Chromosome (linkage group)	Species		
	
	*F. graminearum*	*N. crassa*	*A. nidulans*
1	0	21	0
2	14	0	0
3	0	0	2
4	3	0	0
5		0	0
6		0	0
7		0	0
8			0

### Increased gene duplications and rates of gene evolution within TE clusters

The borders of the three TE clusters were manually defined by inspecting the chromosome browser available at the Chromosome 7 Sequencing Project homepage [28]. The TE clusters span over 88% of the TEs and 40% of the predicted genes on the chromosome (Figure 2). Using the protein sequence clustering tool TRIBE-MCL [29], we grouped chromosome 7 proteins into families. This clustering resulted in the classification of 74 proteins into 32 families of 2 to 4 proteins per family. Percent identity within these families ranged from 28.8% to 100%, with an average of 42.3% identity. Examination of the distribution of gene family members revealed that 62% of the genes were found within the TE clusters while 84% of the families contain at least 1 member within a TE cluster, suggesting that gene duplications are more prevalent within the TE clusters.

The relative rates of evolution between proteins encoded by genes within TE clusters and outside of clusters were compared by computing the evolutionary distance to orthologous proteins in the *N. crassa *and *F. graminearum *genomes. This analysis assumes that all of the orthologous protein pairs diverged at approximately the same time [30]. While the divergence time may not necessarily be the same for all orthologous protein pairs, the mean time of divergence within and outside of the TE clusters should be similar. Putative orthologs to proteins from chromosome 7 were identified from the genomes of *N. crassa *and *F. graminearum *by performing BLASTP searches. Orthologs could be identified in *N. crassa *for 12.7% of the genes within TE clusters and 59.8% outside of the clusters (Table 3). The orthologous protein pairs were aligned over their full length and rate of evolution was inferred by computing Kimura's distance [31] for each alignment. The average distance between orthologous protein pairs was significantly higher between orthologous genes within the TE clusters (0.963) than outside of the TE clusters (0.735), based on a two-tailed Student's *t* test (*P *< 0.05). When this analysis was repeated using the *F. graminearum *genome, we obtained a similar pattern of evolutionary rates (Table 3).

**Table 3:** Number and similarity of putative orthologs between predicted proteins from chromosome 7 and proteins from the *N. crassa *and *F. graminearum*

	Number of genes	*N. crassa*		*F. graminearum*	
		
		Number of proteins with orthologs	D*	Number of proteins with orthologs	D*
Within TE islands	463 (40.2%)	57 (12.3%)	0.963	49 (10.6%)	0.928
Outside of TE islands	688 (59.8%)	211 (30.7)	0.735	223 (32.4%)	0.774
Total	1,151	268		272	

*Average distance of all orthologous protein pairs between the indicated species and chromosome 7; distance (*D*) was computed using Kimura's method. The values were significantly different (*P *< 0.05) based on a two-tailed Student's *t* test.

## Discussion

The recent availability of whole genome shotgun sequences for several filamentous fungi affords us the opportunity for the first time to perform an in-depth analysis of the evolution of the structure of fungal genomes. With the combined BAC-WGS sequence for chromosome 7, we have a more accurate estimate of the true repetitive DNA content of the chromosome as well as a better reference sequence with which to study conservation of synteny. The *M. oryzae *genome is known to be rich in repetitive DNA and several authors have shown that, based on analyses of genomic libraries, TEs are not distributed randomly across the genome, but are tightly clustered. Our analysis of chromosome 7 revealed the presence of three clusters of TEs, two of which are localized near the distal ends of the chromosome while the other is centrally located. The locations of the TEs are positively correlated with recombination rate, a finding that is not predicted by the popular selection models that describe the TE distribution in several other species [15,32]. Several sources of selective pressure are proposed to play a role in the establishment of this type of distribution. The ectopic exchange model states that repetitive DNA promotes deleterious ectopic recombination events. Since negative selection would be reduced in regions of low recombination rate, repetitive DNA would accumulate in regions with low recombination rate. Under the insertion model, TE insertions generally have a deleterious effect on fitness, and are thus likely to be lost from the population. In chromosomal segments that have low recombination rates, deleterious mutations are more likely to be genetically linked to neutral genes, decreasing their negative fitness effect.

Many TEs are known to selectively integrate into specific sequences and this activity, known as site specificity, has also been proposed to play a role in biased TE distribution. Site specificity has been demonstrated for TEs from a wide array of species, including *D. melanogaster*, *Saccharomyces cerevisiae*, *Schizosaccharomyces pombe *and others [33]. In most cases, TEs tend to integrate into intergenic regions. For example, the *S. pombe *TE Tf1 preferentially integrates into a region 100 to 420 bp upstream of translation start sites, though this specificity does not lead to biased TE distribution on a genome level [33]. The *S. cerevisiae *LTR transposons Ty1-Ty4 tend to integrate into regions upstream of genes transcribed by RNA polymerase III (Pol III) and, in the case of Ty3, it has been demonstrated that interactions between Ty3 integration factors and Pol III transcription factors are responsible for this site specificity [34,35]. In *S. cerevisiae*, tRNA genes, which are transcribed by Pol III, dispersed throughout the genome and are not correlated with TE clusters. As shown by Bachman *et al*. [36], the Ty1 element preferentially inserts into upstream regions of specific members of t^Gly ^and t^Thr ^genes, leading to a biased genomic distribution of Ty1. Analysis of predicted tRNA genes in chromosome 7 revealed that the tRNA genes are distributed evenly throughout the chromosome (data not shown). Specificity for tRNA genes could explain the distribution of TEs in the *M. oryzae *genome only if all TE families had the same site specificity, for example, to the same members of tRNA gene families and the targeted family members were also positively correlated with recombination rate. Ty1 elements have also been shown to have specificity for pre-existing LTR transposons [36,37], which would likely result in the formation of TE clusters; however, selection models still are needed to explain the correlation between the distribution of TE clusters and recombination rate.

As is the case in *M. oryzae*, a positive correlation has also been described between DNA transposons and recombination rate in *C. elegans*, although no such correlation was found for retrotransposons [19]. Unlike *C. elegans*, both retrotransposons and DNA transposons show a positive correlation with recombination rate in *M. oryzae*. *C. elegans *is highly inbred, and it has been suggested that this may reduce the effects of recombination rate-based selective pressures on the genome. While *M. oryzae *is a naturally outbreeding species, it is commonly recognized that sexual reproduction occurs only rarely in the field and it is primarily disbursed through asexual propagules. Therefore, the selective pressures imposed by meiotic recombination, though still present, may not be strong enough to force the distribution of TEs into regions of low recombination rate and some force, but not recombination, must be driving the clustering of TEs in the *M. oryzae *genome.

The divergence dates of the species included in this study are difficult to estimate, due to the sparse fossil record for fungi. Based on the phylogeny published by Berbee and Taylor [38], we estimate that the earliest radiation of the Sordariomycetes, which includes *Magnaporthe*, *Fusarium*, and *Neurospora*, was approximately 200 MYA and the divergence of the *Aspergillus *lineage from this group occurred at least 300 MYA. More recent work by Padovan *et al*. [39] suggests that these radiations may be up to 653 MYA and 930 MYA, respectively. Based on these estimates, our initial expectation was that little conservation of synteny at the chromosome level would be evident between these fungi and the early reports of conserved microsynteny between *M. oryzae *and *N. crassa *[20] were presumed to be rare exceptions. However, we found that a significant number of syntenic blocks exist between these species, suggesting that portions of these chromosomes still share common ancestry. The syntenic blocks that we detected contain a large number of intervening non-syntenic genes, many of which did have homologs in the other fungal genomes but to genes in other chromosomal locations, indicating that translocations of small DNA segments containing only one or a few genes occurred over large distances. Perturbations of gene order within blocks were also common and probably resulted from small scale rearrangements.

Nearly all of the syntenic blocks on chromosome 7 occurred in regions that lack TEs and have low recombination rate (Figure 2). Repetitive sequences are known to promote crossing over at non-homologous chromosomal sites, which can lead to chromosomal rearrangements and loss of conserved synteny. Such a correlation has been reported in wheat, where indirect selection due to gene hitchhiking has been hypothesized to be the driving factor in the biased distribution of conserved synteny [40]. Indirect selection is stronger in regions of low recombination, leading to reduced levels of polymorphism, such as insertions and translocations. It is likely that both the presence of repetitive DNA and high recombination rate promote the ectopic recombination events that result in a loss of synteny between species.

Seventy four of the 1,151 predicted proteins on chromosome 7 could be grouped into paralogous families by sequence similarity, of which 64.8% were located within a TE cluster. However, the TE clusters span only 40% of the predicted genes, suggesting that duplicated genes may be more common within TE clusters. If TE clusters lose synteny at a faster rate due to chromosomal rearrangements such as translocations and deletions, then it is reasonable to expect that duplications would also occur with greater frequency in these regions. By comparing protein sequences from chromosome 7 to orthologous proteins from *F. graminearum *and *N. crassa*, we show that evolutionary distance was significantly greater in genes found within the TE clusters. If, on average, the protein pairs within TE clusters diverged from their orthologs at approximately the same time, then this result can be used to infer rate of evolution and we can conclude that genes within the TE clusters are evolving at a faster rate. Therefore, high recombination rate in *M. oryzae *is associated with increased number of gene duplications, increased rate of gene evolution and with loss of synteny, a pattern that has also been described in wheat [40,41].

## Conclusion

Based on the data presented here, we suggest that specific segments of chromosome 7 rapidly lose conserved synteny as a result of rearrangements promoted by increased recombination rate and by the presence of TEs. These rearrangements may also contribute to the formation of new genes by gene duplication, as is suggested by the presence of a higher than expected number of duplicated genes within the TE clusters. Furthermore, within the TE clusters, genes are less likely to have orthologs in *N. crassa *and *F. graminearum *than genes outside of the TE clusters. Based on these findings, we propose that TE clusters are major contributors to the genesis and evolution of new genes in the *M. oryzae *genome.

## Materials and methods

### Data sources

The sequence of chromosome 7 from *M. oryzae*, strain 70-15, was obtained from the chromosome 7 sequencing project [28] (GenBank: CM000230). The whole genome shotgun sequences for *M. oryzae*, *N. crassa*, *F. graminearum*, and *A. nidulans *were obtained from the Broad Institute Fungal Genome Initiative web site [42].

### Annotation

Known repetitive elements were identified and masked from the chromosome 7 sequence with RepeatMasker [24], using a previously prepared database of repetitive elements from the *M. oryzae *genome [43]. Additional annotations (for example, blast searches, expressed sequence tags (EST) alignments) were performed using the masked sequence. The gene prediction program FGENESH (Softberry Corporation, Mount Kisco, NY, USA) trained to predict *M. oryzae *genes [3] was used to identify 1,151 putative gene coding sequences. Recombination rate was measured by identifying the physical locations of nine genetic markers on the chromosome and using them as a basis for delineating eight intervals spanning chromosome 7. The markers, previously anchored to the *M. oryzae *BAC library [9,44], were assigned locations on the chromosome by aligning BAC end sequences to the chromosome sequence. The position of each marker was estimated by taking the average location of all BAC end sequences anchored to both a marker and to the chromosome.

### Analysis of conserved synteny

Blocks of conserved synteny were identified using the algorithm and statistical test implemented in the FISH software package [27]. The FISH algorithm identifies segmental homologies (syntenic blocks) between chromosomal segments either within or between species and uses as input a set of homologous markers. We used as input to the FISH package the results of a BLASTP search in which the predicted proteins from chromosome 7 were used as the query sequences and the proteome set (the set of annotated proteins available from the Broad Institute) for *N. crassa*, *F. graminearum*, and *A. nidulans *were used as blast databases. Thus, one chromosome 7 protein may match more than one protein from another species and these one-to-many mappings were included in the analysis. The default parameters for the FISH software package were used except that the minimal acceptable alignment score (bit score) between protein sequences was raised to 500. Syntenic blocks that contained five or more pairs of genes (*P *< 0.001) were retained. For the purpose of this analysis, protein pairs within statistically significant syntenic blocks were considered to be orthologs.

### Analysis of gene duplications and rate of evolution

Duplicated genes were identified by clustering the protein sequences with TRIBE-MCL. An *E *value cutoff of 1e-5 was used for the initial BLASTP search. A multiple sequence alignment was computed for each cluster using the Muscle program [45] and the mean percentage identity for each cluster was computed as the mean of all pairwise comparisons within the cluster. Putative orthologs to proteins from the *N. crassa *and *F. graminearum *genomes were defined as BLASTP hits with an *E *value smaller than 1e-30 that had no other hits better than 1e-5. Pairwise sequence alignments were performed with the Muscle program and evolutionary distance was calculated using Kimura's method as implemented in the Phylip package [31,46].

## References

[B1] Couch BC Fudal I Lebrun M-H Tharreau D Valent B Van Kim P Notteghem Jl Kohn L Origins of host-specific populations of the blast pathogen *Magnaporthe oryzae *in crop domestication with subsequent expansion of pandemic clones on rice and weeds of rice. Genetics 2005 170 613 630 10.1534/genetics.105.041780 15802503 PMC1450392

[B2] Ou SH Rice Diseases. 1987 Surrey: Commonwealth Mycological Institute

[B3] Dean R Talbot N Ebbole D Farman M Mitchell T Orbach M Thon MR Kulkarni RD Xu J-R Pan H Analysis of the genome sequence of the plant pathogenic fungus *Magnaporthe grisea*, the causal agent of rice blast disease. Nature 2005 434 980 986 10.1038/nature03449 15846337

[B4] Talbot NJ On the trail of a cereal killer: Exploring the biology of *Magnaporthe grisea*. Annu Rev Microbiol 2003 57 177 202 10.1146/annurev.micro.57.030502.090957 14527276

[B5] Valent B Plant disease: Underground life for rice foe. Nature 2004 431 516 10.1038/431516a 15457240

[B6] Bohnert H Fudal I Dioh W Tharreau D Notteghem J Lebrun M A putative polyketide synthase/peptide synthetase from *Magnaporthe grisea *signals pathogen attack to resistant rice. Plant Cell 2004 16 2499 2513 10.1105/tpc.104.022715 15319478 PMC520948

[B7] Farman ML Eto Y Nakao T Tosa Y Nakayashiki H Mayama S Leong SA Analysis of the structure of the AVR1-CO39 avirulence locus in virulent rice-infecting isolates of *Magnaporthe grisea*. Mol Plant-Microbe Interact 2002 15 6 16 11843304 10.1094/MPMI.2002.15.1.6

[B8] Kang S Lebrun MH Farrall L Valent B Gain of virulence caused by insertion of a Pot3 transposon in a *Magnaporthe grisea *avirulence gene. Mol Plant-Microbe Interact 2001 14 671 674 11332731 10.1094/MPMI.2001.14.5.671

[B9] Zhu H Choi SD Johnston AK Wing RA Dean RA A large-insert (130 kbp) bacterial artificial chromosome library of the rice blast fungus *Magnaporthe grisea*: Genome analysis, contig assembly, and gene cloning. Fungal Genet Biol 1997 21 337 347 10.1006/fgbi.1997.0996 9290247

[B10] Thon MR Martin SL Goff S Wing RA Dean RA BAC end sequences and a physical map reveal transposable element content and clustering patterns in the genome of *Magnaporthe grisea*. Fungal Genet Biol 2004 41 657 666 10.1016/j.fgb.2004.02.003 15275661

[B11] Nitta N Farman ML Leong SA Genome organization of *Magnaporthe grisea*: Integration of genetic maps, clustering of transposable elements and identification of genome duplications and rearrangements. Theor Appl Genet 1997 95 20 32 10.1007/s001220050528

[B12] Nishimura M Nakamura S Hayashi N Asakawa S Shimizu N Kaku H Hasebe A Kawasaki S Construction of a BAC library of the rice blast fungus *Magnaporthe grisea *and finding specific genome regions in which its transposons tend to cluster. Biosci Biotechnol Biochem 1998 62 1515 1521 10.1271/bbb.62.1515 9757557

[B13] Daboussi MJ Fungal transposable elements: Generators of diversity and genetic tools. J Genet 1996 75 325 339

[B14] Daboussi MJ Fungal transposable elements and genome evolution. Genetica 1997 100 253 260 10.1023/A:1018354200997 9440278

[B15] Bartolomé C Maside X Charlesworth B On the abundance and distribution of transposable elements in the genome of *Drosophila melanogaster*. Mol Biol Evol 2002 19 926 937 12032249 10.1093/oxfordjournals.molbev.a004150

[B16] The Arabidopsis Genome Initiative Analysis of the genome sequence of the flowering plant *Arabidopsis thaliana*. Nature 2000 408 796 10.1038/35048692 11130711

[B17] Dasilva C Hadji H Ozouf-costaz C Nicaud S Jaillon O Weissenbach J Crollius HR Remarkable compartmentalization of transposable elements and pseudogenes in the heterochromatin of the *Tetraodon nigroviridis *genome. Proc Natl Acad Sci USA 2002 99 13636 13641 10.1073/pnas.202284199 12368471 PMC129727

[B18] Rizzon C Marais G Gouy M Biemont C Recombination rate and the distribution of transposable elements in the *Drosophila melanogaster *genome. Genome Res 2002 12 400 407 Article published online before print in February 2002 10.1101/gr.210802 11875027 PMC155295

[B19] Duret L Marais G Biemont C Transposons but not retrotransposons are located preferentially in regions of high recombination rate in *Caenorhabditis elegans*. Genetics 2000 156 1661 1669 11102365 10.1093/genetics/156.4.1661 PMC1461346

[B20] Hamer L Pan HQ Adachi K Orbach MJ Page a Ramamurthy L Woessner JP Regions of microsynteny in *Magnaporthe grisea *and *Neurospora crassa*. Fungal Genet Biol 2001 33 137 143 10.1006/fgbi.2001.1286 11456466

[B21] Talbot NJ Salch YP Ma M Hamer JE Karyotypic variation within clonal lineages of the rice blast fungus, *Magnaporthe grisea*. Appl Environ Microbiol 1993 59 585 593 16348876 10.1128/aem.59.2.585-593.1993 PMC202148

[B22] Hedges SB The origin and evolution of model organisms. Nat Rev Genet 2002 3 838 849 10.1038/nrg929 12415314

[B23] Galagan J Calvo S Borkovich K Selker E Read N Jaffe D Fitzhugh W Ma L Smirnov S Purcell S The genome sequence of the filamentous fungus *Neurospora crassa*. Nature 2003 422 859 868 10.1038/nature01554 12712197

[B24] RepeatMasker Open-3.0. http://www.repeatmasker.org

[B25] Davis CR Kempainen RR Srodes MS Mcclung CR Correlation of the Physical and Genetic Maps of the Centromeric Region of the Right Arm of Linkage Group III of *Neurospora crassa*. Genetics 1994 136 1297 1306 7912215 10.1093/genetics/136.4.1297 PMC1205910

[B26] Farman ML Leong Sa Chromosome walking to the AVR1-CO39 avirulence gene of *Magnaporthe grisea*: Discrepancy between the physical and genetic maps. Genetics 1998 150 1049 1058 9799257 10.1093/genetics/150.3.1049 PMC1460382

[B27] Calabrese PP Chakravarty S Vision TJ Fast Identification and statistical evaluation of segmental homologies in comparative maps. Bioinformatics 2003 19 74 80 10.1093/bioinformatics/btg1008 12855440

[B28] The Chromosome 7 Sequencing Project Homepage http://www.fungalgenomics.ncsu.edu/chromosome_seven/

[B29] Enright AJ Van Dongen S Ouzounis CA An efficient algorithm for large-scale detection of protein families. Nucleic Acids Res 2002 30 1575 1584 10.1093/nar/30.7.1575 11917018 PMC101833

[B30] Hirsh AE Fraser HB Protein dispensability and rate of evolution. Nature 2001 411 1046 1049 10.1038/35082561 11429604

[B31] Kimura M The neutral theory of molecular evolution. 1983 Cambridge: Cambridge University Press

[B32] Wright SI Agrawal N Bureau TE Effects of recombination rate and gene density on transposable element distributions in *Arabidopsis thaliana*. Genome Res 2003 13 1897 1903 12902382 10.1101/gr.1281503 PMC403781

[B33] Behrens R Hayles J Nurse P Fission yeast retrotransposon Tf1 integration is targeted to 5' ends of open reading frames. Nucleic Acids Res 2000 28 4709 4716 10.1093/nar/28.23.4709 11095681 PMC115174

[B34] Kirchner J Connolly C Sandmeyer S Requirement of RNA polymerase III transcription factors for *in vitro* position-specific integration of a retroviruslike element. Science 1995 267 1488 1491 7878467 10.1126/science.7878467

[B35] Connolly C Sandmeyer S RNA polymerase III interferes with Ty3 integration. FEBS Lett 1997 405 305 311 10.1016/S0014-5793(97)00200-7 9108309

[B36] Bachman N Eby Y Boeke J Local definition of Ty1 target preference by long terminal repeats and clustered tRNA genes. Genome Res 2004 14 1232 1247 10.1101/gr.2052904 15197163 PMC442138

[B37] Ji H Moore D Blomberg M Braiterman L Voytas D Natsoulis G Boeke J Hotspots for unselected Ty1 transposition events on yeast chromosome III are near tRNA genes and LTR sequences. Cell 1993 73 1007 1018 10.1016/0092-8674(93)90278-X 8388781

[B38] Berbee ML Taylor JW McLaughlin D, McLaughlin E, Lemke P Fungal molecular evolution: gene trees and geologic time. The Mycota 2001 7 part B Berlin, Germany: Springer-Verlag 229 245

[B39] Padovan AC Sanson GF Brunstein A Briones MR Fungi evolution revisited: application of the penalized likelihood method to a bayesian fungal phylogeny provides a new perspective on phylogenetic relationships and divergence dates of Ascomycota groups. J Mol Evol 2005 60 726 735 10.1007/s00239-004-0164-y 15909224

[B40] Akhunov ED Goodyear AW Geng S Qi LL Echalier B Gill BS Miftahudin Gustafson JP Lazo G Chao SM The organization and rate of evolution of wheat genomes are correlated with recombination rates along chromosome arms. Genome Res 2003 13 753 763 10.1101/gr.808603 12695326 PMC430889

[B41] Akhunov ED Akhunova AR Linkiewicz AM Dubcovsky J Hummel D Lazo G Chao S Anderson OD David J Qi L Synteny perturbations between wheat homoeologous chromosomes caused by locus duplications and deletions correlate with recombination rates. Proc Natl Acad Sci USA 2003 100 10836 10841 10.1073/pnas.1934431100 12960374 PMC196889

[B42] The Broad Institute Fungal Genome Initiative http://www.broad.mit.edu/annotation/fungi/fgi/

[B43] Martin SL Blackmon BP Rajagopalan R Houfek TD Sceeles RG Denn SO Mitchell TK Brown DE Wing RA Dean RA MagnaportheDB: a federated solution for integrating physical and genetic map data with BAC end derived sequences for the rice blast fungus *Magnaporthe grisea*. Nucleic Acids Res 2002 30 121 124 10.1093/nar/30.1.121 11752272 PMC99159

[B44] Zhu H Blackmon BP Sasinowski M Dean RA Physical map and organization of chromosome 7 in the rice blast fungus, *Magnaporthe grisea*. Genome Res 1999 9 739 750 10447509 PMC310806

[B45] Edgar RC MUSCLE: multiple sequence alignment with high accuracy and high throughput. Nucl Acids Res 2004 32 1792 1797 10.1093/nar/gkh340 15034147 PMC390337

[B46] Felsenstein J PHYLIP - Phylogeny Inference Package (Version 3.2). Cladistics 1989 5 164 166

